# Purpose in life as an asset for well-being and a protective factor against depression in adolescents

**DOI:** 10.3389/fpsyg.2023.1250279

**Published:** 2023-09-27

**Authors:** Barbara Barcaccia, Alessandro Couyoumdjian, Micaela Di Consiglio, Carolina Papa, Uberta Ganucci Cancellieri, Matti Cervin

**Affiliations:** ^1^Department of Psychology, Sapienza University of Rome, Rome, Italy; ^2^Associazione di Psicologia Cognitiva APC and Scuola di Psicoterapia Cognitiva Srl SPC, Rome, Italy; ^3^University for Foreigners “Dante Alighieri” of Reggio Calabria, Reggio Calabria, Italy; ^4^Department of Clinical Sciences, Lund University, Lund, Sweden

**Keywords:** purpose in life, well-being, stress, anxiety, depression, self-reassurance, adolescents

## Abstract

Purpose in life, which is a central component of the eudaimonic paradigm of well-being, has been sparsely examined in adolescence. This is unfortunate as adolescence is characterised by identity development and is a key period for the onset of mental disorders. To inform future research on well-being and purpose in life in adolescents, we drew factors from several fields of research, including mental health and psychological factors, and explored which factors were most strongly associated with purpose in life. Data were collected in a sample of 444 Italian adolescents (M_age_ = 16.30 [SD = 1.50], range: 14 to 20 years; 58% girls) and associations with mental health (stress, anxiety, depression, anger), psychological traits (mindfulness, self-hate, self-inadequacy, self-reassurance, isolation), and sociodemographic variables (age, sex, place of birth) were examined. Regression, dominance, and network analyses indicated that a stronger sense of purpose in life was associated with lower depressive symptoms, higher levels of self-reassurance, and being born in Italy. Our findings suggest that purpose in life is an important asset for well-being in adolescents and may protect against depression. Future longitudinal and/or experimental research should examine the potential protective role of purpose in life in relation to adolescent depression and how self-reassurance and sociodemographic factors (e.g., immigrant background) are involved.

## Introduction

Adolescence is a crucial time for establishing the bases of well-being and mental health. Indeed, most mental disorders onset during adolescence, with the peak age being 14.5 years, and almost half of all children develop a mental disorder before reaching adulthood (Solmi et al., 2022). The most common adolescent mental health problems are depression and anxiety (Kessler et al., 2007). A plethora of research has shown that such symptoms negatively impact overall development, social relationships, and academic performance and result in substantial personal, social and economic costs (Gore et al., 2011; Auerbach et al., 2014; Copeland et al., 2014; Hetrick et al., 2016; Monroe et al., 2019). The other side of the coin, that of adolescent well-being, has been less explored.

The hedonistic and the eudaimonic approaches are the two main theoretical frameworks of well-being. The hedonistic approach emphasises positive emotions and subjective well-being, whereas the eudaimonic approach emphasises psychological well-being in the form of a “flourishing” life, with scope and meaning, and living a life coherent with one’s true self (Diener, 1984; Ryan and Deci, 2012). In the eudaimonic framework, feeling a purpose in life is a key element (Ryff and Keyes, 1995; Deci and Ryan, 2008; Ryff and Singer, 2008), while it is less pronounced in the hedonistic paradigm (Malin et al., 2017). Ryff and Keyes (1995) describe six facets of psychological well-being: (1) “self-acceptance” (a positive evaluation of oneself and one’s past), (2) “personal growth,” (3) “positive relations with others,” (4) “environmental mastery” (the ability to effectively manage one’s life and the surrounding world), (5) “autonomy” (a sense of self-determination), and (6) “purpose in life” (the belief that one’s life is meaningful and purposeful).

Purpose can be defined as “a long-term, forward-looking intention to accomplish aims that are meaningful to oneself as well as impacting the world beyond oneself” (Malin et al., 2017, p. 1201). Purpose also implies the setting of goals and a sense of hope for the future, and rather than being dictated by the society, family or peers, these values and goals emerge from the individual’s own motivation and aspiration (Bundick, 2011). Yet, while it is a subjective experience, it also goes beyond the self and includes other people’s well-being and motivations to contribute to the community (Bronk et al., 2009; Bronk, 2014). Furthermore, purpose in life can be considered an aim that inspires and guides the choice of goals (McKnight and Kashdan, 2009), which can be attainable or not, but are nonetheless pursued (Bronk and Finch, 2010).

A growing body of literature has begun to shed light on the crucial role that having a sense of purpose plays in promoting well-being, as well as protecting individuals against the risks of psychopathology. Moreover, research has shown that individuals with a greater purpose in life have better psycho-physical health throughout the life span (Kim et al., 2019; Pfund and Lewis, 2020).

Previous studies in adults and older adults have found that the presence of purpose in life and of conceptually similar constructs, such as meaning in life, is negatively associated with symptoms of depression and anxiety, and plays a beneficial role in counteracting them (Pearson et al., 2015; Yek et al., 2017; Salt et al., 2018; Laird et al., 2019; Crego et al., 2021; Mei et al., 2021; Yager and Kay, 2023). Indeed, a recent meta-analysis showed significant negative associations of purpose in life with depression and anxiety in both healthy and clinical populations (Boreham and Schutte, 2023). Loneliness, which is a correlate of depressive and anxious symptomatology, is negatively associated with general well-being and with purpose in life (Bondevik and Skogstad, 2000). In particular, social exclusion has been found to negatively impact the degree to which individuals experience a sense of purpose (Stillman and Baumeister, 2009).

Regarding the population of adolescents, a longitudinal study (Chen and Cheng, 2020) on high school students showed that increases in purpose predicted increased satisfaction with life and decreased depressive symptoms among both girls and boys. Along these lines, Chen et al. (2021) found that the presence of meaning in life was negatively associated with symptoms of depression and anxiety, and in another study, a greater goal orientation was associated with lower symptoms of depression (Askeland et al., 2020). Recently, found that greater purpose exploration and commitment was associated with lower depression and higher hope, prosocial tendency, self-efficacy and life satisfaction in high-school students. Overall, the presence of meaning and purpose in life has been linked to better psychological adjustment variables and also to improved academic performance in youth (Pizzolato et al., 2011; Abdul Kadir and Mohd, 2021).

While several studies have examined how purpose in life is associated with feelings of depression, anxiety and loneliness, little is known about associations with another important human emotion, anger. It has been hypothesised that higher levels of anger may be linked to a greater sense of purpose, as anger might enhance motivation to strive towards a challenging goal (Tamir, 2016; Wilson and Hill, 2023). Some studies indicate that anger is negatively associated with psychological well-being (Diong and Bishop, 1999), but specific associations with purpose in life have rarely been examined.

Dispositional mindfulness, i.e., the capacity of paying attention to the present moment in a non-judgmental and non-reactive fashion (Kabat-Zinn, 2015) is thought to positively affect purpose in life. Although relatively few studies have examined this directly, some evidence of a positive association has been published (Crego et al., 2021).

Purpose in life has also been linked to the ways in which a person relates to oneself, where self-warmth, self-reassurance and self-compassion are of relevance and entail “a positive and warm attitude for the self that allows acceptance, compassion and understanding of flaws and failures as part of the human condition” (Castilho et al., 2015, p. 154; Castilho et al., 2015, p. 154). Specifically, self-compassion has been defined as being warm and reassuring toward oneself at times of suffering and failure, rather than spending time and energy in self-deprecation or harsh self-criticism (Germer and Neff, 2013). Experiencing warm and reassuring feelings toward oneself has been linked to better well-being, including to a sense of purpose in life (Neely et al., 2009). Furthermore, individuals who are more other-forgiving and less revengeful experience greater well-being, including purpose in life (Lawler-Row and Piferi, 2006), and lower psychopathology (Barcaccia et al., 2022) also among adolescents (Barcaccia et al., 2019). Conversely, feelings of unforgiveness negatively affect the capacity of finding meaning and purpose (Akhtar et al., 2017).

Most research on purpose in life has been conducted with adults, whereas the number of studies on adolescents is relatively small. The present work aims at filling this gap by delving into the multifaceted relations between purpose in life and other important psychological variables in adolescents, with a specific focus on its potential as a protective mechanism against psychopathology, namely depression. Indeed, adolescence represents a key period during which the foundations of well-being are built: it has been suggested that when adolescents live their lives with a purpose, they experience psychological well-being (Witten et al., 2019). During adolescence, the individual starts thinking about who they are and who they want to be in the future (Hill et al., 2021; Ratner et al., 2023). The adolescent years are also characterised by increased capacity for abstract thinking, which allows for reflecting about meaning and purpose in life, including setting proximal and distant goals for one’s own life (Sawyer et al., 2012). Research suggests that purpose in life during adolescence promotes adaptive growth (Pfund and Lewis, 2020), may play a role in identity development (Burrow and Hill, 2011) and improves overall well-being (Bronk and Finch, 2010; Ratner et al., 2023). Similar to findings in adults, purpose in life in adolescence is positively associated with positive affect, subjective well-being, and life satisfaction (Burrow and Hill, 2011; Bronk, 2014; Pfund and Lewis, 2020). Purpose has also been linked to desirable personality characteristics that may contribute to healthy development, such as compassion, gratitude, generosity and conscientiousness (Burrow and Hill, 2011; Malin et al., 2017). Furthermore, adolescents with a strong sense of purpose tend to do better academically (Yeager and Bundick, 2009; Yeager et al., 2014).

To inform research on how well-being in adolescents can be understood and improved, the aim of this study is to conduct a theory-informed exploration of purpose in life in adolescence. We will draw factors from several fields of research, including mental health (i.e., symptoms of depression, stress, anxiety and anger), psychological traits (i.e., mindfulness, attitudes towards oneself), isolation, and sociodemographic information (e.g., age, gender and background). The study is conducted in an exploratory fashion, but we expect that several factors will be uniquely associated with purpose in life and that the strongest association will emerge in relation to depressive symptoms.

## Method

### Participants and procedure

The sample comprised 444 adolescents with a mean age of 16.30 (SD = 1.50, range: 14 to 20). A majority were girls (*n* = 257, 57.9%) and the rest boys (*n* = 187, 42.1%) and a vast majority were born in Italy (*n* = 418, 94.1%). The study is part of a larger research project assessing a number of dimensions associated with adolescent mental health and well-being, which has been conducted across high schools located in various Italian regions. Data collection for the present study took place in five different high schools, and an opportunity sampling procedure without exclusion criteria was used. The headmasters of five high schools were notified via e-mail, where the purpose of the study was explained. All headmasters approved that the students at their schools could complete a battery of questionnaires during regular class hours. Students were subsequently invited to take part in the study, and written informed consent was obtained by the parents/guardians of underage students (<18), whereas those who were of legal age (≥18) signed their own informed consent. All potential participants were assured that the collection of data was anonymous and voluntary. After written consent was obtained, participants received a link to an electronic survey on the online platform LimeSurvey, where they completed the questionnaires. The collection of data was obtained in a single session in their classrooms, where they completed the online survey in a quiet classroom environment, with a research assistant and a teacher present. Participants needed approximately 35 min to complete the battery. The study was conducted according to the Declaration of Helsinki and approved by the schools’ boards and the ethics committee of Roma Tre University.

### Measures

**The Life Engagement Test** (LET; Scheier et al., 2006) is a 6-item questionnaire that evaluates the extent to which an individual has a sense of meaning and purpose in life. Respondents rate the extent to which they agree with each statement on a 5-point Likert scale, from 1 (strongly disagree) to 5 (strongly agree). The six items are: “There is not enough purpose in my life” (reverse scored), “To me, the things I do are all worthwhile,” “Most of what I do seems trivial and unimportant to me” (reverse scored), “I value my activities a lot,” “I do not care very much about the things I do” (reverse scored), “I have lots of reasons for living.” After reverse scoring, all scores are summed, with higher scores indicating greater purpose in life. The internal consistency of the LET in the present study was adequate (*α* = 0.76, *ω* = 0.76).

The reverse translation procedure from English into Italian was carried out by two independent translators. The LET was translated into Italian by a bilingual clinical psychologist, experienced in research on adolescents and familiar with this instrument of measure. Afterwards it was back-translated into English by a professional translator. Points of divergence were resolved by conference (Brislin, 1980).

**The Depression Anxiety and Stress Scale-21** (DASS-21; Lovibond and Lovibond, 1995a; Lovibond and Lovibond, 1995b; Bottesi et al., 2015) is a 21-item questionnaire evaluating levels of depression, anxiety and stress over the previous week. Respondents rate how much each statement applies to them on a 4-point Likert scale, from 0 (never) to 3 (almost always). Each subscale is composed of seven items. The internal consistency of all DASS-21 subscales was good in the present sample (depression, *α* = 0.89, *ω* = 0.89; anxiety, *α* = 0.83, *ω* = 0.84; stress, *α* = 0.85, *ω* = 0.85).

**The Forms of Self-Criticizing and Self-Reassuring Scale** (FSCRS; Gilbert et al., 2004; Petrocchi and Couyoumdjian, 2016) is a 22-item questionnaire assessing self-criticism in response to failures or setbacks. Two subscales measure two different facets of self-criticism, “inadequate self” and “hated self,” whereas the third subscale measures the capability of being reassuring and supportive to oneself. Respondents rate how much each statement applies to them on a 5-point Likert scale (from 0 = “not at all like me” to 4 = “extremely like me”). The internal consistency of the three FSCRS scales was good to excellent (Self-hate, *α* = 0.82, *ω* = 0.83; Self-inadequacy, *α* = 0.91, *ω* = 0.91; Self-reassurance, *α* = 0.86, *ω* = 0.86).

**Child and Adolescent Mindfulness Measure** (CAMM; Greco et al., 2011; I-CAMM; Ristallo et al., 2016) is a 10-item questionnaire assessing mindfulness in children and adolescents. Respondents rate how much each statement applies to them on a 5-point Likert scale ranging from 0 (never true) to 5 (always true). In the current dataset the internal consistency of the I-CAMM was adequate (*α* = 0.77, *ω* = 0.78).

**State-Trait Anger Expression Inventory-2 Child and Adolescent** (STAXI-2C/*A*; Brunner and Spielberger, 2009; Lonigro et al., 2015) is a 35-item questionnaire measuring anger in children and adolescents across five subscales: trait anger, state anger, anger expression-in, anger expression-out, and anger control. For the purpose of this study, we only used the trait anger subscale (10 items), which assesses chronic feelings of anger as opposed to state anger, which is more fluctuating. The scale had adequate internal consistency in the present sample (*α* = 0.79, *ω* = 0.79).

**Trait Forgivingness Scale** (TFS; Berry et al., 2005; Barcaccia et al., 2018) measures dispositional forgiveness and includes 7 statements, to which participants rate how much each applies to them on a 5-point Likert scale (1 = strongly disagree, 5 = strongly agree). The scale had adequate internal consistency in the present sample (*α* = 0.72, *ω* = 0.72).

**Self-Compassion Scale (SCS)** (Neff, 2003; Petrocchi et al., 2014) is a 26-item scale that measures several aspects related to self-compassion. It includes six subscales: self-kindness, common humanity, mindfulness, self- judgment, isolation and over-identification. In the present study, we only included the isolation subscale (4 items) which showed good internal consistency (*α* = 0.86, *ω* = 0.87).

### Statistical analysis

The sample size depended on the number of participating schools and how many adolescents consented to participate and thus was not predetermined. However, the resulting sample of 444 provided us with adequate statistical power (0.84) to detect even a small effect (quantified as a regression coefficient) in a multiple linear regression model. Similarly, the sample size provided us with reasonably adequate statistical power (0.69) to detect a group difference of moderate size even for the most unbalanced group comparison (born in versus outside of Italy). Associations between purpose in life and the other study variables were analysed in several ways. First, we used t-tests to compare groups (boys/girls, being born in/outside of Italy). Second, we examined the zero-order Pearson correlations between purpose in life and age and all the mental health and psychological trait variables. To parse out unique associations, we conducted a linear regression model with purpose in life as the dependent variable and all other variables as independent variables. Multicollinearity was examined by estimating the Variance Inflation Factor (VIF) for each independent variable and values above 5 were considered to be potentially concerning. The regression analysis was followed-up by dominance analysis, in which all possible subset models of independent variables were tested to examine the degree of unique variation in the dependent variable accounted for by each independent variable. To further explore unique associations among variables, we estimated a network using all study variables. In a network, each variable is depicted as a circle and each unique association as a line. Unique associations were estimated as partial correlations using partial correlations using the R library *BGGM*. Because we included a mix of continuous and binary variables, we used a semi-parametric copula model based on ranked likelihood to estimate the partial correlations, which can range from −1 to +1. All network associations were estimated using 5,000 posterior samples and 95% credible intervals were used to control for false positive rate. The network analysis was conducted because it is a conservative method to explore associations between variables (partial correlations instead of regression coefficients) and because it does not assume a causal relation between variables. Because the study was largely exploratory, an alpha level of 0.05 and credible intervals of 95% were used as indicators of statistical significance.

## Results

**Figure 1:**
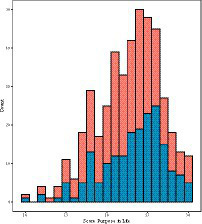
Histogram showing the distribution of purpose in life scores for boys and girls, with the upper bars indicating the count for girls.

Participants varied in their purpose in life (see Figure 1 for histograms for boys and girls separately). Boys (M = 23.24 [3.92]) reported significantly higher purpose in life than girls (M=22.39 [3.91]), but the effect size was small (*t*[442] = 2.26, *p* = 0.02, Cohen’s *d* = 0.22). Homogeneity of variance could not be assumed in this model, but the Welch *t*-test statistic yielded very similar results (*t*[400.6] = 2.26, *p* = 0.02). Age was not significantly correlated with purpose in life (*r* = −0.06, *p* = 0.25). Participants born in Italy (M = 22.95 [3.91]) reported significantly higher purpose in life than those born outside of Italy (M = 19.58 [2.91]) and the effect size was large (*t*[442] = 4.32, *p* < 0.001, Cohen’s *d* = 0.87) and homogeneity of variance could be assumed.

The zero-order correlations between purpose in life and stress, anxiety, depression, anger, self-hate, self-inadequacy, self-reassurance, mindfulness, forgiveness, and isolation alongside means and standard deviations are in Table 1. To examine normality of the included continuous variables reported in Table 1, we computed the Skewness and Kurtosis values for each variable. All Skewness values were in the range of −0.49 to 1.05 and all Kurtosis values in the range of −0.70 to 0.50, indicating no apparent violation of normality. Several variables were significantly and weakly to moderately correlated with purpose in life, with the strongest associations emerging in relation to depression (negative association), self-hate (negative association) and self-reassurance (positive association).

**Table 1:** Means and standard deviations and zero-order correlations among study variables.

	M (SD)	Stress	Anxiety	Depression	Anger	Self-hate	Self-inadequacy	Self-reassurance	Mindful-ness	Forgiveness	Isolation
Purpose in life	21.58 (4.10)	−0.35**	−0.37**	−0.53**	−0.23**	−0.51**	−0.39**	0.51**	−0.32**	0.02	−0.41**
Stress	9.52 (4.19)	–	0.70**	0.76**	0.52**	0.50**	0.59**	−0.38**	0.54**	−0.18**	0.52**
Anxiety	23.95 (7.24)		–	0.66**	0.40**	0.53**	0.51**	−0.37**	0.53**	−0.15**	0.49**
Depression	26.07 (5.92)			–	0.43**	0.62**	0.63**	−0.51**	0.53**	−0.10*	0.62**
Anger	28.00 (6.69)				–	0.36**	0.49**	−0.24**	0.48**	−0.28**	0.49**
Self-hate	9.52 (4.19)					–	0.72**	−0.64**	0.48**	−0.28**	0.49**
Self-inadequacy	23.95 (7.24)						–	−0.58**	0.63**	−0.07	0.73**
Self-reassurance	26.07 (5.92)							–	−0.32**	0.09	−0.50**
Mindfulness	28.01 (6.69)								–	−0.10*	0.59**
Forgiveness	21.15 (5.10)									–	−0.10*
Isolation	11.73 (4.07)										–

To get a better sense of which variables were most uniquely associated with purpose in life, we conducted a linear regression model with purpose in life as the dependent variable and all other measured variables including age, sex, and being born in versus outside of Italy, as independent variables. The model was statistically significant (*p* < 0.001) and explained 41.0% of the variation in purpose in life. No independent variable had a VIF above 5 (the highest VIF was 3.27 for depressive symptoms) and the Durbin–Watson test yielded a value of 1.05, indicating no severe autocorrelation. Results of the regression model are presented in Table 2. Higher scores on depression, self-hate, and self-inadequacy were significantly associated with lower purpose in life scores. Further, being a girl, being born in Italy, and scoring higher on the self-reassurance scale were associated with higher purpose in life scores.

**Table 2:** Results from the linear regression model with purpose in life as the dependent variable.

	*B*	Standardized beta	*p*
Stress	0.09	0.11	0.11
Anxiety	−0.02	−0.02	0.68
Depression	**−0.28**	**−0.37**	**< 0.001**
Anger	−0.05	−0.04	0.40
Self-hate	**−0.17**	**−0.18**	**< 0.01**
Self-inadequacy	**0.08**	**0.15**	**0.03**
Self-reassurance	**0.19**	**0.28**	**< 0.001**
Mindfulness	−0.04	−0.06	0.22
Forgiveness	−0.04	−0.04	0.37
Isolation	−0.08	−0.08	0.18
Age (years)	0.02	0.01	0.86
Sex (boy = 1, girl = 0)	**−0.81**	**−0.10**	**0.01**
Born in Italy (yes = 1, no = 0)	**2.97**	**0.18**	**< 0.001**

Statistically significant associations are highlighted in bold.

Dominance analysis showed that depression explained most unique variance in purpose in life (9.5%) followed by self-reassurance (9.1%), self-hate (7.0%), being born in Italy (3.6%), isolation (3.3%), self-inadequacy (2.6%), anxiety (2.3%), stress (2.1%), mindfulness (1.6%), being a boy (0.7%), anger (0.7%), forgiveness (0.1%), and age (0.0%).

**Figure 2:**
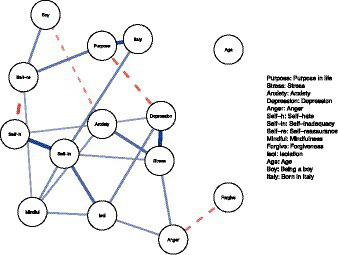
Associative network structure of study variables. Each circle represents a variable and each line a statistically significant partial correlation. Wider lines indicate stronger partial correlations. Solid lines indicate a statistically significant positive partial correlation and dashed lines indicate a statistically significant negative partial correlation. Variables are placed according to their associations with each other, with more strongly associated variables being places closely together and variables with many associations with other variables being places centrally.

Last, we estimated a network to capture the full associative structure of all study variables. The network structure is presented in Figure 2. In line with the regression and dominance analyses, purpose in life was uniquely linked to depression (partial correlation = −0.29), self-reassurance (partial correlation = 0.28), and being born in Italy (partial correlation = 0.46).

## Discussion

Few studies have examined purpose in life in adolescents, which is a key component in the eudaimonic framework of well-being. The present study was conducted to guide future research about how to best understand feelings of purpose in adolescents and which research avenues may be most important to pursue. In order to achieve these goals, we selected a range of variables that have been theoretically and/or empirically linked to purpose in life in previous research. A focus on purpose in life, and in extension well-being in adolescence (Witten et al., 2019) is important to better understand the development and maintenance of adolescent mental health and illness and how adolescents can achieve their full potential and flourish (Ciarrochi et al., 2013). Considering that most mental disorders typically begin during adolescence (Jones, 2013; Solmi et al., 2022), this is a crucial period for prevention of mental health conditions and promotion of well-being.

In line with our expectations, depressive symptoms and purpose in life were strongly and negatively associated. Our results confirm previous findings on the role of purpose in life, which prior research has found to be strongly associated with depression, both in adults (Pearson et al., 2015; Salt et al., 2018; Crego et al., 2021), and in adolescents: increases in purpose predicted increased satisfaction with life and decreased depression in high school students (Chen and Cheng, 2020); meaning in life was negatively associated with depression and anxiety in middle and high school students (Chen et al., 2021); greater goal orientation was associated with lower depression in adolescents aged 16–19 (Askeland et al., 2020). In summary, purpose in life (and conceptually similar constructs) has been linked to better overall psychological adjustment variables, and our findings confirm that having a sense of purpose in life plays a crucial role in enhancing adolescents’ well-being and protecting them from depression.

Of note, depressive symptoms and purpose in life were uniquely associated in all statistical models. Loss of interest and low mood are core symptoms of depression, and many depressed individuals experience hopelessness (American Psychiatric Association, APA, 2013). As engagement, hope and a future-oriented outlook are integral to purpose in life, the strong link to depressive symptoms is not surprising. It can be speculated that participants with a greater purpose in life had clearer goals, were more hopeful and motivated to reach their goals, and engaged more in personal meaningful activities. In fact, engaging in valued and meaningful activities is a form of behavioral activation, which is known to protect against depression (Hooker et al., 2020).

Adolescents who had greater purpose in life were also less critical about themselves and more compassionate, with the clearest association emerging in relation to self-reassurance: purpose and self-reassurance were uniquely associated in both the regression and network models. Self-reassurance refers to the ability to be reassuring, compassionate and encouraging towards oneself at times of setbacks and failure (Germer and Neff, 2013; Petrocchi et al., 2019). It is possible that this trait protects against self-criticism and self-hate, which in turn may impede efforts to engage in life and one’s own goals. In the present study, self-reassurance was also uniquely linked to self-hate, supporting this hypothesis. It is also possible that having purpose means being more enterprising, i.e., more used to actively engage in achieving goals, and at the same time more accustomed to the possibility of failure, seen as part of human condition. Therefore, those adolescents whose lives are characterised by purpose and commitment, are also more capable of considering setbacks and failures as normal hindrances occurring in everyone’s life, and may be more capable of reassuring themselves when things go wrong. Our results shed light on the crucial role that purpose in life plays in shaping psychological well-being of adolescents: having a sense of purpose may not only decrease the risk of depression but also contribute to overall well-being and quality of life, confirming findings from previous studies on the beneficial role of purpose in life (Pizzolato et al., 2011; Askeland et al., 2020; Chen and Cheng, 2020; Abdul Kadir and Mohd, 2021; Chen et al., 2021), and suggesting the potential benefits of promoting a sense of purpose as a preventive measure against depression and as a means of promoting well-being among adolescents.

Further, there was no significant association between being a boy and purpose in life in the full network of variables. However, being a boy was closely linked to higher self-reassurance, which in turn was linked to purpose, indicating that self-reassurance is important to explain the differences between boys and girls in the present study.

In line with our exploratory stance, all available information was analysed in relation to purpose in life. Regarding sociodemographic information, few variables were available (age, gender, and birth place). A surprising but interesting finding was the clear association between being born in or outside of Italy, where adolescents being born in Italy reported a substantially stronger feeling of purpose in life. Interestingly, in the network, birthplace was uniquely linked only to purpose in life and stronger feelings of self-inadequacy, indicating that this variable was of particular relevance to purpose in life. Our findings regarding birthplace are partially in line with previous studies suggesting that well-being may be lower in immigrant adolescents when compared to their native peers (Borraccino et al., 2018; Alivernini et al., 2020), but are not consistent with previous data indicating that immigrant youth, when compared to their native counterparts, show higher well-being (Sam et al., 2008; Van Geel and Vedder, 2010; Dimitrova, 2011; Güngör and Perdu, 2017).

This study has a number of limitations that must be acknowledged. Firstly, our results are based on data collected from a convenience sample, which may impact representativity. Secondly, all data were self-reported. Thirdly, the cross-sectional nature of the study does not allow for causal inference, which could be better investigated in future experimental or longitudinal studies.

The present study was conducted in an exploratory fashion to increase knowledge about purpose in life in adolescents. In line with our hypothesis, a clear link emerged in relation to depression. Because of the cross-sectional nature of the study, future research needs to examine how purpose and depressive symptoms are linked longitudinally. While it is reasonable to assume that purpose protects against depressive symptoms, depression is a heterogeneous and complex disorder with a complex aetiology and it is possible or even likely that the relation between purpose in life and depressive symptoms is reciprocal. We also found a clear association between self-reassurance and purpose in life, which is intriguing as this suggests that the capacity of being encouraging and compassionate towards oneself may be in important factor to consider in prevention and health promotion during adolescence, but again longitudinal or experimental studies are needed. Last, we found a strong association between having an immigrant background and experiencing lower purpose in life. While this association was very clear in the present study, it is only partially in line with previous research, and thus should be further examined.

## Data availability statement

The raw data supporting the conclusions of this article will be made available by the authors, without undue reservation.

## Ethics statement

The studies involving humans were approved by Ethics Committee of Roma Tre University, Rome, Italy. The studies were conducted in accordance with the local legislation and institutional requirements. Written informed consent was obtained from the parents/guardians of underage students, whereas those who were of legal age signed their own informed consent.

## Author contributions

BB, MC, and AC contributed to conception and design of the study and wrote the first draft of the manuscript. MDC and CP organised the database and contributed to the data interpretation and editing of the manuscript. UGC supervised the study and edited the manuscript. MC performed the statistical analysis and wrote the results section. All authors contributed to the article and approved the submitted version.

## Conflict of interest

The authors declare that the research was conducted in the absence of any commercial or financial relationships that could be construed as a potential conflict of interest.

## Publisher’s note

All claims expressed in this article are solely those of the authors and do not necessarily represent those of their affiliated organizations, or those of the publisher, the editors and the reviewers. Any product that may be evaluated in this article, or claim that may be made by its manufacturer, is not guaranteed or endorsed by the publisher.
